# Next-Generation Sequencing Reveals a Very Low Prevalence of Deleterious Mutations of Homologous Recombination Repair Genes and Homologous Recombination Deficiency in Ovarian Clear Cell Carcinoma

**DOI:** 10.3389/fonc.2021.798173

**Published:** 2022-01-12

**Authors:** Hangqi Liu, Zhiwen Zhang, Longyun Chen, Junyi Pang, Huanwen Wu, Zhiyong Liang

**Affiliations:** Department of Pathology, State Key Laboratory of Complex Severe and Rare Disease, Molecular Pathology Research Center, Peking Union Medical College Hospital, Chinese Academy of Medical Sciences and Peking Union Medical College, Beijing, China

**Keywords:** ovarian clear cell carcinoma, homologous recombination repair gene, homologous recombination deficiency, molecular classification, next-generation sequencing

## Abstract

Ovarian clear cell carcinoma (OCCC) is aggressive and drug-resistant. The prevalence of homologous recombination repair (HRR) gene mutations and homologous recombination deficiency (HRD) remains largely unknown. It is also not clear whether the commonly used molecular-based classification for endometrial carcinoma (EC) is potentially applicable in OCCC. In this study, surgically resected samples were collected from 44 patients with OCCC. Genomic alterations were determined using next-generation sequencing. HRD was estimated by genomic instability. Of 44 patients with OCCC, two (4.5%) harbored likely pathogenic mutations in HRR genes. Notably, no pathogenic or likely pathogenic mutations were found in *BRCA1/2*. *A* total of 24 variants of uncertain significance (VUS) in HRR-related genes occurred in 18 (40.9%) patients. HRD was observed in only one case (2.3%). In addition, *TP53* mutation and microsatellite instability-high (MSI-H) were identified in three patients (6.8%) and in one patient (2.3%), respectively. *TP53* mutation was significantly associated with disease-free survival and overall survival. No *POLE* mutations were found. In conclusion, our results revealed a very low prevalence of HRR gene mutations and HRD in OCCC. Moreover, *TP53* mutations and MSI-H are uncommon, while *POLE* mutations are extremely rare in OCCC. Our findings indicate that the evaluation of HRR gene mutations, HRD status, *POLE* mutations, and MSI-H may have limited clinical significance for OCCC treatment and prognostic stratification.

## Introduction

Ovarian cancer (OC) is one of the most devastating and malignant gynecological tumors. Ovarian clear cell carcinoma (OCCC) is a distinctive histological subtype of OC that accounts for 4%–25% of all OC, with most cases being diagnosed at an advanced stage (1). Currently, the management approach for advanced OCCC is a combination of debulking surgery and platinum chemotherapy (2). However, OCCC poses a significant clinical challenge as they are less sensitive to standard platinum-based chemotherapy, with poorer prognosis than other major histological subtypes of OC (3). Moreover, the majority of OCCC patients experience tumor recurrence and therapeutic strategies are limited especially in late-stage or recurrent tumor (4).

Although the mechanisms of carcinogenesis and chemoresistance of OC remain quite unclear, molecular changes have been widely investigated. It has been well known that genomic alterations in *BRCA1* and *BRCA2* render OC deficient in the homologous recombination repair (HRR) pathway (5). Germline or somatic mutations in other HRR-related genes can also cause homologous recombination deficiency (HRD) (6). OC with HRD including *BRCA1/2* mutations is sensitive to poly (ADP-ribose) polymerase (PARP) inhibitors, which induce synthetic lethality *via* inhibition of DNA single-stranded break repair mechanisms and ultimately lead to cell death (5). The National Comprehensive Cancer Network (NCCN) Guidelines recommend implementing *BRCA* and HRD testing in patients with recurrence or metastasis OC (7). Recent studies have revealed that almost half of the high-grade serous ovarian cancer (HGSOC) have HRD (6, 8). However, limited information about HRR pathway gene abnormalities and HRD scores has been obtained in OCCC.

Another important advance in molecular diagnostics of gynecological malignancies is the four distinct molecular subclasses of endometrial carcinoma (EC) proposed by The Cancer Genome Atlas (TCGA) (9). These include the *POLE*/ultramutated (*POLE*), microsatellite-instable/hypermutated (MSI), copy-number-low/p53-wild-type (CNL), and copy-number-high/p53-mutant (CNH) (9). These results could serve as valuable tools in prognostic significance and treatment implications (10). It has been recognized that OCCC shares molecular and histological similarities with EC, with atypical endometriosis supposed to be the precursor lesion of both diseases (11). Furthermore, the MSI status, a well-known predictive marker for immune checkpoint inhibitors, was recommended to be detected by the NCCN Guidelines in patients with recurrent or advanced OC (7). Herein, we performed similar molecular characterization including *POLE* mutation, *TP53* mutation, and MSI status to explore the mutation status and potential prognostic and therapeutic role of these markers in OCCC.

## Materials and Methods

### Patients and Clinicopathological Characteristics

This retrospective study used data collected from the archives of the Department of Pathology at Peking Union Medical College Hospital (PUMCH) between December 2018 and June 2020. A total of 44 patients with a proven diagnosis of OCCC from PUMCH were enrolled in this study. All hematoxylin-eosin-stained specimens were re-confirmed by two specialist pathologists. Histological diagnosis was assigned based on the World Health Organization classification scheme. Clinicopathological features including age at diagnosis, the International Federation of Obstetrics and Gynecology (FIGO) stage, residual tumor after debulking surgery, adjuvant chemotherapy, neoadjuvant therapy, tumor recurrence, and follow-up time were recorded. Ethical approval was granted by the Institutional Review Board of PUMCH.

### Sample Preparation, Next-Generation Sequencing, and Data Processing

Eight 5-μm tumor slices cut from formalin-fixed paraffin-embedded (FFPE) samples were used for genomic DNA extraction. The HRR gene combination detection library preparation kit (Amoy Diagnostics, Xiamen, China) was used for DNA library construction following the manufacturer’s protocol. Genomic DNA libraries were applied to an HRR 30-gene panel (including 19 HRR genes: *ATM*, *ATR*, *BRCA1*, *BRCA2*, *BARD1*, *CHEK1*, *CHEK2*, *FANCA*, *FANCL*, *PALB2*, *RAD51B*, *RAD51C*, *RAD51D*, *RAD54L*, *CDK12*, *NBN*, *PPP2R2A*, *BRIP1*, and *MRE11A*, and 11 genetic and therapy-related genes: *BRAF*, *CDH1*, *ESR1*, *KRAS*, *TP53*, *NRAS*, *PIK3CA*, *HOXB13*, *ERBB2*, *PTEN*, and *STK11*). Then, DNA sequencing was performed on the NextSeq500 Illumina platform (Illumina, San Diego, CA, USA). The average depth was 1,000×, and the effective sequencing depth was greater than 300×. The proportion of Q30 bases was ≥75%. Several types of mutations including single-nucleotide polymorphisms (SNPs), Indels, and hot-spot mutations of the oncogenic genes were detected. Sequencing data were analyzed by Sequencing Data Analysis Software (Amoy Diagnostics, Xiamen, China); variant results were evaluated based on the sequencing quality and confidence of the locus, and only variants with VAF ≥ 3% were retained. For patients with HRR gene mutations detected in tumor tissue, tissues adjacent to the tumor were also tested to distinguish between germline and somatic mutations. Deleterious and suspected deleterious gene mutations were scrutinized and interpreted according to the American College of Medical Genetics and Genomics (ACMG) guidelines and/or in ClinVar (12). The Genbank accession numbers of reference sequences are listed in **Supplementary Table 1**.

### HRD Score Analysis

Genomic DNA from FFPE samples was extracted for library construction and captured with AmoyDx^®^ HRD-focus panel to screen coding sequence (CDS) regions for *BRCA1* and *BRCA2* genes and 24,000 SNPs for HRD calling. Sequencing was performed on the Illumina Nextseq500 platform, where the effective depth for *BRCA* gene and SNPs loci were greater than 200× and 100×, respectively. Sequencing data were analyzed using Amoy Sequencing Data Analysis Software (Amoy Diagnostics, Xiamen, China). The HRD score was calculated by the sum of three types of genomic unstable events, which included loss of heterozygosity (LOH), telomeric allelic imbalance (TAI), and large-scale state transition (LST). HRD-positive was defined by either *BRCA1/2* pathogenic/likely pathogenic (P/LP) mutation or HRD score ≥ 42 (13). Pathogenic and LP mutations annotation of *BRCA1/2* were classified according to ACMG guidelines (12).

### Molecular Classification

To explore the frequency of *POLE* mutation, *TP53* mutation, and MSI status in patients with OCCC, we performed sequencing analysis with the AmoyDx^®^ BPTM panel. The sequencing results of *POLE*, *TP53* mutations, and MSI status from the FFPE samples were analyzed comprehensively. OCCC patients were categorized into the appropriate molecular subtypes, including four groups: *POLE*-mutant, *TP53*-wild-type, *TP53*-mutant, and microsatellite instability-high (MSI-H). Gene mutations were annotated based on the ACMG guidelines (12). MSI status was evaluated based on the integrated assessment of genome-wide 55 microsatellite loci. If the number of unstable microsatellite loci was more than 15% of the total number of loci, the sample would be determined as MSI-H; otherwise, it would be determined as microsatellite stable (MSS). Lynch syndrome (LS) was subsequently confirmed by a 5-gene panel associated with LS in MSI-H patients by using targeted capture and NGS on the Illumina Nextseq500 platform.

### Immunohistochemical Staining

Immunohistochemical (IHC) staining for p53 and mismatch repair (MMR) proteins including MSH2, MSH6, MLH1, and PMS2 were performed on 4-μm FFPE tissue sections using a BenchMark ULTRA autostainer, version 12.3 (Ventana Medical Systems, USA). Four MMR and p53 protein antibodies (Beijing Zhongshan Golden Bridge Biotechnology, China) were used according to the manufacturer’s recommendations. Immunohistochemical staining was considered absent when there was a complete loss of nuclear expression by the tumor cells compared to the internal positive controls.

### Statistical Analysis

Disease-free survival (DFS) was defined as the time interval between the date of surgery and the date of recurrence or the last follow-up. Overall survival (OS) was calculated from the date of the surgery to either the date of death from any cause or the last follow-up. Survival analysis was performed using the Kaplan–Meier survival curves, and comparisons between the groups were assessed by the log-rank test. SPSS version 24 (SPSS Inc., Chicago, IL, USA) was used for statistical analyses. *p*-value <0.05 was considered statistically significant.

## Results

### Clinicopathologic Features and Survival

Clinicopathologic characteristics of the 44 patients are summarized in **Table 1**. The patient’s age ranged from 30 to 72 years, with a median age of 51.5 years. Half of the patients were diagnosed at FIGO stage I (22 cases). The number of patients with FIGO stage II, III, and IV was 10 (22.7%), 9 (20.5%), and 3 (6.8%), respectively. Forty-one women (93.2%) had the residual tumor with sizes ≤ 2 cm after debulking surgery. All patients underwent surgery and received postoperative adjuvant treatment. Moreover, 15 patients (34.1%) received neoadjuvant therapy before surgery. After a median follow-up time of 20 months (range, 1–28 months), 11 patients (25.0%) developed recurrent disease and 3 of them died of OCCC. The median DFS was 17.8 months and the median OS was 19.7 months in our cohort. The FIGO stage III/IV was found to be significantly associated with worse DFS (*p* = 0.0107, **Supplementary Figure 1A**).

**Table 1:** Clinical characteristics of forty-four OCCC patients.

Clinical characteristics	Cases (%)
Age (years)	
≤51	22 (50.0)
>51	22 (50.0)
FIGO Stage	
I	22 (50.0)
II	10 (22.7)
III	9 (20.5)
IV	3 (6.8)
Residual tumor (cm)	
≤2	41 (93.2)
>2	3 (6.8)
Adjuvant chemotherapy	
Yes	44 (100.0)
No	0 (0)
Neoadjuvant therapy	
Yes	15 (34.1)
No	29 (65.9)
Tumor recurrence	
Yes	11(25.0)
No	33 (75.0)
DFS mean (months)	
≤17.8	17 (38.6)
>17.8	27 (61.4)
OS mean (months)	
≤19.7	21 (47.7)
>19.7	23 (52.3)

### Variants of Uncertain Significance and Pathogenic/Likely Pathogenic Alterations in HRR-Related Genes and Other Genes

**Figure 1:**
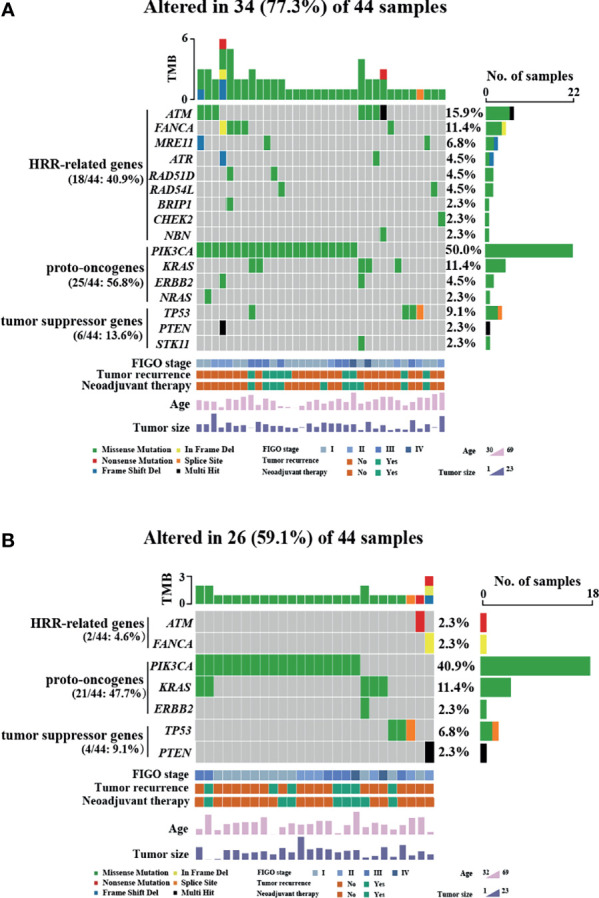
Variants of HRR-related genes and other genes in patients with OCCC. Mutational profile of both pathogenic/likely pathogenic variants and VUS **(A)** and pathogenic/likely pathogenic variants **(B)**. Each column represents one case, and rows represent an individual gene. The patients are presented in decreasing order based on the number of patients in whom a gene is mutated. The right panel indicates the frequency of gene mutations. Mutation types are indicated by different colors. Gray denotes an absence of mutations. Only variants with a VAF of >3% are shown. Clinicopathological features including age at diagnosis, FIGO stage, tumor size, neoadjuvant therapy, and tumor recurrence are annotated below the oncoprint.

**Figure 2:**
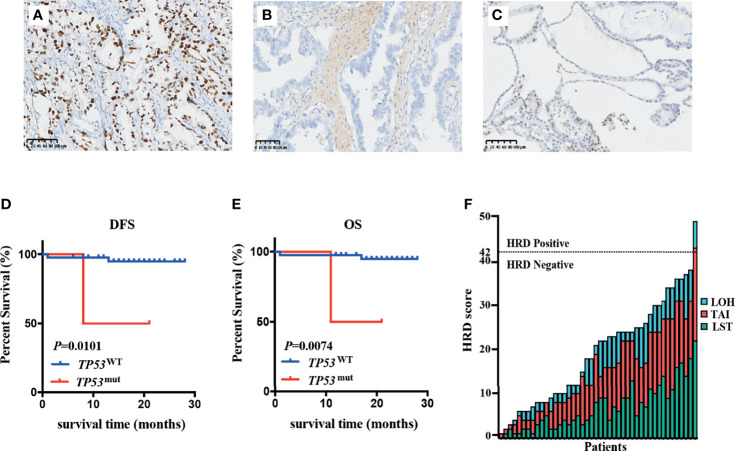
Relationship between *TP53* mutation status and patients’ prognosis and HRD status in OCCC patients. **(A–C)** Immunohistochemistry was performed on tumors with *TP53* mutation, showing intense and diffuse p53 immunostaining for likely pathogenic missense mutation **(A)**, completely absent p53 immunostaining for splicing mutation **(B)**, and rare p53-positive cells with VUS **(C)**. **(D, E)** Kaplan–Meier survival curves based on *TP53* immunohistochemistry results. Patients with *TP53* mutations had poor DFS (*p* = 0.0101) **(D)** and OS (*p* = 0.0074) **(E)**. *p*-values are calculated using the log-rank test. **(F)** HRD score in patients with OCCC. The columns and rows represent individual patient and HRD scores, respectively.

HRR-related gene alterations were determined in all patients (**Figure 1**). Overall, variants of HRR-related genes and other genes were identified in 34 of 44 (77.3%) patients (**Figure 1A**). A total of 24 variants of uncertain significance (VUS) occurred in 9 HRR-related genes in 18 patients (40.9%), including *ATM*, *FANCA*, *MRE11*, *ATR*, *BRIP1*, *RAD51D*, *RAD54L*, *CHEK2*, and *NBN*. Notably, *ATM* was the most common mutation gene at 15.9%, followed by *FANCA* and *MRE11*, which were mutated in 11.4% and 6.8% of the tumors tested, respectively. In addition to HRR pathway genes, the panel also detected mutations in four oncogenes commonly found in OC, including *PIK3CA* (50.0%), *KRAS* (11.4%), *ERBB2* (4.5%), and *NARS* (2.3%). Alterations of *TP53*, *PTEN*, and *STK11* tumor suppressor genes were also detected and the mutation frequencies of these three genes were 9.1%, 2.3%, and 2.3%, respectively. Among carcinomas with *TP53* mutations, two LP alterations c.503A>G (p.His168Arg) and c.832C>T (p.Pro278Ser) were found in two tumor samples and they were all positive for p53 staining (**Figure 2A**). One case with a splice site at the intron 6 (c.673-2A>G) displayed a complete lack of p53 expression in IHC staining (**Figure 2B**). The tumor harboring VUS c.391A>C (p.Asn131His) was stained negative with IHC (**Figure 2C**). We also performed survival analyses to determine the prognostic value of *TP53* mutations in patients with OCCC. The Kaplan–Meier plots revealed that *TP53* mutations were correlated with worse DFS (*p* = 0.0101, **Figure 2D**) and OS (*p* = 0.0074, **Figure 2E**). Additionally, the distribution of P/LP mutations and VUS depending on FIGO stage and age were investigated (**Supplementary Figures 1B**–**G**). The older age (>50 years) at diagnosis was significantly associated with the frequency of HRR-related gene mutations (*p* = 0.004, **Supplementary Figure 1B**). However, no correlation between age and proto-oncogenes or tumor suppressor genes mutation frequency was observed (**Supplementary Figures 1C, D**). The FIGO stage was not associated with the frequency of P/LP mutations and VUS in HRR-related genes and other genes (**Supplementary Figures 1E**–**G**).

### Pathogenic/Likely Pathogenic Alterations in HRR-Related Genes and Other Genes

Next, we assessed pathogenic/likely pathogenic (P/LP) mutation of HRR-related genes and other genes in OCCC. After excluding VUS, a total of 26 samples from 44 patients (59.1%) had P/LP alterations (**Figure 1B**). Only 2 individuals (4.5%) harbored LP mutations in the HRR pathway genes. One case showed LP mutation in *ATM*: c.5944C>T (p.Gln1982*) and the other case showed LP mutation in *FANCA*: c.3788_3790delTCT (p.Phe1263del). Variants in three oncogenes were found in 21 of 44 (47.7%) patients, which included *PIK3CA* (40.9%), *KRAS* (11.4%), and *ERBB2* (2.3%). Among the tumor suppressor genes that were mutated in our cohort, we identified that the frequency of *TP53* and *PTEN* mutations were 6.8% and 2.3%, respectively. The distribution of P/LP alterations according to FIGO stage and age is demonstrated in **Supplementary Figure 2**. A very low frequency of deleterious mutations of HRR-related genes was detected either in the different age categories (**Supplementary Figure 2A**) or FIGO stage (**Supplementary Figure 2D**). There was no correlation between P/LP mutation frequency and FIGO stage or age at diagnosis (**Supplementary Figures 2A**–**F**). Additionally, the FIGO stage of patients showed no significant difference between the younger (≤60 years) and older age group (>60 years) (**Supplementary Figure 2G**).

### Evaluation of HRD Score

The genomic scars of HRD were observed by its three components (LOH, TAI, and LST), which could be quantified separately or combined as a measurement of HRD phenotype. To investigate the HRD status in patients with OCCC, the HRD score was calculated by unweighted sum of LOH, TAI and LST scores. The HRD characteristics of individual patients including its three components’ scores (LOH, TAI, and LST) are displayed in **Figure 2F**. HRD scores ranged from 1 to 49. A score of 42 was defined as the threshold of HRD. Of 44 patients, only one patient (2.3%) had a high HRD score (≥42). *PIK3CA* His1047Arg missense mutation and VUS in *ATM* and *MRE11* were detected in the patients with high HRD (**Supplementary Table 2**).

### *POLE* Mutation, *TP53* Mutation, and MSI Status in OCCC

**Figure 3:**
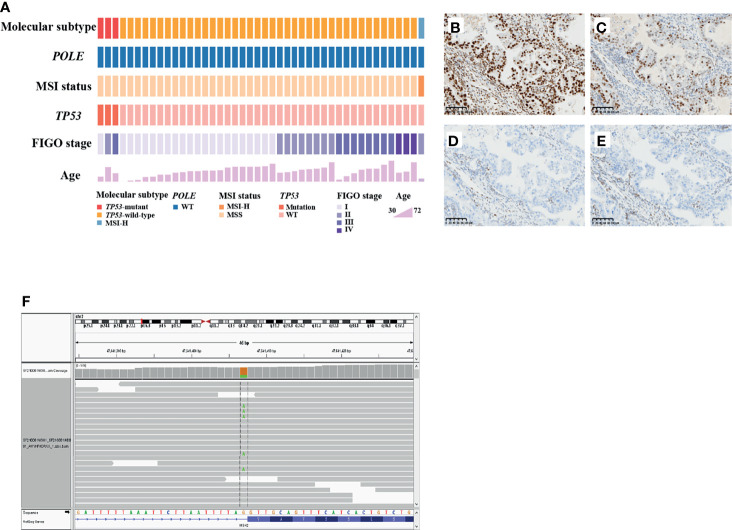
*TP53* mutation, *POLE* mutation, and MSI status in patients with OCCC. **(A)** Tumors were stratified into three groups: *TP53*-wild-type subgroup, *TP53*-mutant subgroup and microsatellite instability-high (MSI)-H subgroup. The MSI-H case showed intact expression of MLH1 **(B)** and PMS2 **(C)**, but loss of MSH2 **(D)** or MSH6 **(E)** expression. Positively staining stromal cells were seen in the background as an internal control. **(F)** Lynch syndrome was confirmed by the presence of *MSH2* pathogenic germline variant in the MSI-H patient.

Considering the similarities between OCCC and endometriosis, we applied the EC molecular-based classification for OCCC to explore the frequency of *POLE* mutation, *TP53* mutation, and MSI status in patients with OCCC. The most prevalent subgroups were the *TP53*-wild-type subgroup (90.9%), while the *TP53*-mutant subgroup (6.8%) and the MSI-H subgroup (2.3%) were less common (**Figure 3A**). *POLE* mutations were not detected in any patients. For the *TP53*-mutant subgroup, three LP *TP53* variants were identified: c.673-2A>G (intron6), c.503A>G (p.His168Arg), and c.832C>T (p.Pro278Ser). Of note, among the 44 OCCC patients analyzed, only one case (2.3%) was MSI-H and the remaining 43 were MSS. MMR proteins’ (MSH2, MSH6, MLH1, and PMS2) IHC staining was performed in tumor tissue from the MSI-H patient, which revealed deficient mismatch repair (dMMR) protein status (**Figures 3B–E**). Furthermore, the MSI-H patient harbored a splice site mutation in *MSH2* (NM_000251.2:intron4:c.793-1G>A) and thus was diagnosed as Lynch syndrome (LS) (**Figure 3F**). Molecular features and subgroups assigned to each patient are reported in detail in **Supplementary Table 2**.

## Discussion

Given that the current standard of care in OC is optimal debulking surgery followed by platinum-based chemotherapy, the observed platinum resistance of OCCCs, particularly in the advanced FIGO stage, poses a considerable clinical challenge. Deciphering alterations in OCCC molecular expression is important, not only for understanding the pathogenesis of this rare disease, but also for exploring effective and alternative personalized treatments for patients who are chemotherapeutic resistant.

In this retrospective investigation, we discovered a high frequency of VUS in HRR-related genes in OCCC patients. Two decades of *BRCA1* and *BRCA2* testing and research have led to a significant decrease in *BRCA* VUS rates, which are lower than most other genes (14). High rates of VUS in other HRR genes are probably due to limited data available for interpretation compared to *BRCA1* and *BRCA2*. Excluding VUS, we observed a low prevalence of HRR gene mutations in OCCC. Previous studies have reported that the prevalence of *BRCA* mutations varies among different EOC subtypes, with the highest prevalence of 20%–25% reported for the high-grade serous subtype (15). Compared with HGSOC, *BRCA* mutations were reported in <10% of the endometrioid subtype and with very low frequency in clear cell subtype (6.3%) (16). One study of 1,195 women with OC investigated the pathogenic mutation in a select set of HRR pathway genes. In the subset of 28 patients with OCCC, there were 2 (7.1%) with mutations in *BRCA1*, one (3.6%) with mutations in *BRCA2*, and 3 (10.7%) with mutations in other HRR genes (17), whereas Pennington et al. found that 26% of OCCC in their cohort harbored HRR-related mutations (18). Similarly, approximately one-third of OCCC in a Japanese cohort was reported to harbor germline and somatic mutations in HRR-related genes (19). Moreover, Hjortkjaer et al. reported that nearly half of the OCCC exhibited the BRCAness phenotype, including having *BRCA1* hypermethylation or no BRCA1 protein expression or mutation in 18 HRR genes (20). These data suggest that a significant proportion of OCCC have HRR gene alterations and may be susceptible to PARP inhibitor therapy. The different prevalence in HRR-related gene mutations between our data and previous studies may be due to limited sample size and cohort differences.

The distribution of gene mutations according to age and FIGO stage was also investigated. Our result showed that the prevalence of P/LP and VUS in HRR-related gene mutations was higher in the older (>50 years) than in the younger (≤50 years) age group. However, a previous study has reported that the presence of germline mutations in the homologous recombination pathway was correlated with early age in prostate cancer patients (21). The discrepancy between our study and previous literature may be related to the different cancer types. Furthermore, Kentaro et al. have investigated the correlation between FIGO stage and HRR-related gene mutations of each histological subtype in 207 ovarian cancer samples (19). All endometrioid carcinomas harboring HRR-related gene mutations were diagnosed at FIGO stage I, whereas there was no difference in HRR-related gene mutation frequency per stage in HGSOC and OCCC. Similarly, our results showed no correlation between the mutation status of HRR-related genes and FIGO stage. Further analysis will be needed to elucidate the clinical significance of HRR-related gene alterations in OCCC in larger cohorts.

In OC, the loss of one specific DNA repair mechanism, the HRR mechanism, results in HRD and is associated with genomic instability, genetic perturbations (duplications, deletions, and translocations of DNA) and ultimately tumor growth (5). In the TCGA project, approximately half of the HGSOC cases are reported to have HRD due to an HRR pathway abnormality (22). Cunningham et al. identified 5 (27.8%) women with HRD, with a higher proportion observed in HGSOC (44.7%) and high-grade endometrioid subtype (37.0%) (23). Angel et al. reported that HGSOC had far higher HRD scores than OCCC, as over 80% of HGSOC cases scored ≥ 42, suggesting that HRD plays a less significant role in OCCC pathogenesis (24). Indeed, low HRD frequency was observed in the present study. This may be affected by different cohorts and pathohistological characteristics. The clinical significance of HRD in different histological types of OC still needs to be further evaluated. Additionally, the HRD-high patient harbored a *PIK3CA* pathogenic missense mutation and VUS in *ATM* and *MRE11*, which needed further analyses to investigate pathogenicity.

Benefiting from well-studied molecular characteristics of OC, the treatment of this disease has made great breakthroughs with the development of precision diagnosis and targeted therapeutic strategies. Currently, PARP inhibitors, such as olaparib, rucaparib, and niraparib, which improve progression-free survival, particularly in patients harboring *BRCA* mutations, are approved by the Food and Drug Administration (FDA) and European Medicine Agency (EMA) for the treatment of OC (25). Notably, to date, PARP inhibitor clinical trials have focused on HGSOC, and endometrioid EOC, rather than on clear cell subtype. Only a minority of OCCC patients have the potential to benefit from PARP inhibitor therapy due to the low frequency of *BRCA* mutations (26). In this study, we demonstrated a very low prevalence of deleterious mutations in HRR genes and HRD in OCCC, indicating that the current molecularly targeted therapies may have little or no effect on OCCC. The most frequently mutated gene identified in this study was *PIK3CA*. Somatic mutation of *PIK3CA* increases phosphatidylinositol 3-kinase (PI3K) activity and activates the downstream AKT signaling pathway, which is one of the major signaling pathways for regulating cell proliferation and survival (27). In support of our results, somatic activating *PIK3CA* mutations were frequently observed in OCCC (at 40%–50%) in the previous study (28). These data indicated that *PIK3CA* may play a much more important role than HRR gene mutations and HRD status in the pathogenesis of OCCC (28), and inhibitors of the PI3K pathway might serve as potential therapeutic options. In addition to *PIK3CA*, several other putative gene alterations are linked to aid novel treatment strategies in OC. Identification of *ERBB2* amplification from the recent work provided other potential therapeutic targets for OC patients (29).

Considering OCCC may possess similar molecular characteristics as EC, we applied the EC molecular-based classification to explore the frequency of *POLE* mutation, *TP53* mutation, and MSI status in OCCC. TCGA demonstrated four categories based on genomic characterization of mutation profiles: *POLE* (7%), MSI (28%), CNL (39%), and CNH (26%) (9). Compared with EC, our data revealed that the prevalence of MSI-H, *POLE*, and *TP53* mutations was very low in OCCC. Contrary to our results, an increasing number of studies have shown that the proportion of MSI-H in OCCC is high (30). Kathy et al. detected about 21% of their OCCC patients exhibited MSI-H (31). Howitt et al. examined a total 30 cases of OCCC and found MSI-H or loss of MMR protein expression was detected in 3 tumors (10.0%) (32). In the study of Bennett et al., 6 samples (5.5%) exhibited loss of MMR proteins in 109 OCCC patients (33). The different prevalence in MSI status between our data and previous study may be related to the testing methodology or inherent differences. Additionally, OCCC is a more common tumor in the ovary to be associated with LS compared with other subtypes (30). Indeed, the MSI-H patient was diagnosed as LS in the present study. Aberrant p53 expression in OCCC has been reported in 7% of OCCC and correlated with worse DFS (34). Moreover, *TP53* mutations occurring in about 10% of OCCC have been associated with unfavorable outcome (35). These data suggest that *TP53* mutation is a potentially useful biomarker to identify patients with OCCC at higher risk for adverse clinical outcomes. In addition, no pathogenic *POLE* mutations were found in the present study. Likewise, previous work demonstrated that none of the cases harbored *POLE* mutations in any of the 90 OCCC patients (34). Based on our findings, the value of *POLE* analysis in OCCC subtyping and prognostic assessment seems to be limited. The discovery of biomarkers that can refine the prognostic stratification of patients with OCCC, beyond the currently used molecular classification for EC explored before, is necessary.

In conclusion, our results indicated that HRR gene mutations and HRD were rare in OCCC. We also revealed that *TP53* mutations, *POLE* mutations, and MSI-H were all uncommon in OCCC. Our findings indicate the prognosis value of *TP53* mutation in patients with OCCC. However, the evaluation of HRR gene mutations, HRD status, *POLE* mutations, and MSI-H status may have limited clinical significance for OCCC treatment and prognostic stratification. Nevertheless, our findings still require further validation in large cohorts to accurately determine the prevalence of HRR-related gene mutations in OCCC. Moreover, identification of other targetable molecular variations in OCCC is warranted, which could make patients eligible for additional experimental and standard therapies.

## Data Availability Statement

The datasets presented in this study can be found in online repositories. The names of the repository/repositories and accession number(s) can be found in the article/**Supplementary Material**.

## Ethics Statement

The studies involving human participants were reviewed and approved by the Institutional Review Board of Peking Union Medical College Hospital. The patients provided their written informed consent to participate in this study.

## Author Contributions

HL analyzed data and completed the manuscript. ZZ, LC, and JP collected specimen and clinical information. HW and ZL designed and supervised the study. All authors contributed to the article and approved the submitted version.

## Funding

This work was supported by the National Natural Science Foundation of China (Project No.82072749) to ZL and the National Natural Science Foundation of China (Project No.82072747) to HW.

## Conflict of Interest

The authors declare that the research was conducted in the absence of any commercial or financial relationships that could be construed as a potential conflict of interest.

## Publisher’s Note

All claims expressed in this article are solely those of the authors and do not necessarily represent those of their affiliated organizations, or those of the publisher, the editors and the reviewers. Any product that may be evaluated in this article, or claim that may be made by its manufacturer, is not guaranteed or endorsed by the publisher.

## Supplementary Material

Supplementary Figure 1 Distribution of VUS and P/LP alterations according to age and FIGO stage in OCCC. **(A)** Association between the FIGO stage and survival in patients with OCCC. The FIGO stage III/IV was significantly associated with worse DFS (*p* = 0.0107). *P* values were calculated using the log-rank test. The frequency of VUS and P/LP mutations in HRR-related genes **(B)**, proto-oncogenes **(C)** or tumor suppressor genes **(D)** according to different age categories (≤ 50 years and > 50 years). The older age (> 50 years) at diagnosis was associated with frequency of HRR-related genes mutations (*p* = 0.004). The frequency of VUS and P/LP mutations in HRR-related genes **(E)**, proto-oncogenes **(F)** or tumor suppressor genes **(G)** according to FIGO stage (FIGO I/II and FIGO III/IV). **(B–G)** were calculated by the Wilcoxon test.

Supplementary Figure 2 Distribution of P/LP alterations according to age and FIGO stage in OCCC. The frequency of P/LP mutations in HRR-related genes **(A)**, proto-oncogenes **(B)** or tumor suppressor genes **(C)** according to different age categories (≤ 50 years and > 50 years). The frequency of P/LP mutations in HRR-related genes **(D)**, proto-oncogenes **(E)** or tumor suppressor genes **(F)** according to FIGO stage (FIGO I/II and FIGO III/IV). **(A–F)** were calculated by the Wilcoxon test. **(G)** The correlation between FIGO stage and different age categories (≤ 60 years and > 60 years). *P* values were calculated by the Kruskal-Wallis test.
