# Supplementary figures and images for: Next-Generation Sequencing Reveals a Very Low Prevalence of Deleterious Mutations of Homologous Recombination Repair Genes and Homologous Recombination Deficiency in Ovarian Clear Cell Carcinoma
Source: Front Oncol. 2022 Jan 12;11:798173. doi: 10.3389/fonc.2021.798173 (PMC8791260; doi:10.3389/fonc.2021.798173)

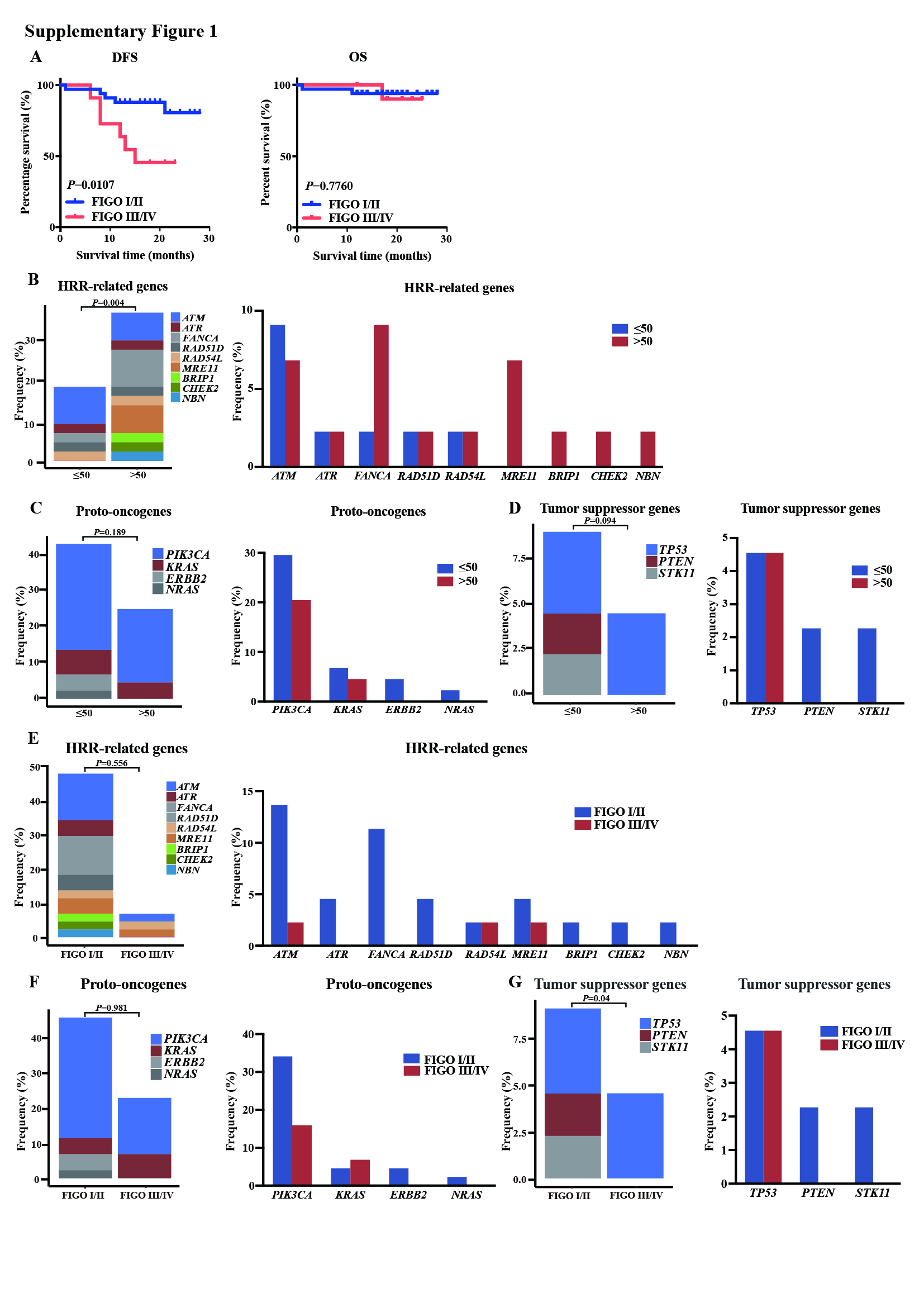

Supplement: Supplementary Figure 1 — Distribution of VUS and P/LP alterations according to age and FIGO stage in OCCC. (A) Association between the FIGO stage and survival in patients with OCCC. The FIGO stage III/IV was significantly associated with worse DFS (p = 0.0107). P values were calculated using the log-rank test. The frequency of VUS and P/LP mutations in HRR-related genes (B), proto-oncogenes (C) or tumor suppressor genes (D) according to different age categories (≤ 50 years and > 50 years). The older age (> 50 years) at diagnosis was associated with frequency of HRR-related genes mutations (p = 0.004). The frequency of VUS and P/LP mutations in HRR-related genes (E), proto-oncogenes (F) or tumor suppressor genes (G) according to FIGO stage (FIGO I/II and FIGO III/IV). (B–G) were calculated by the Wilcoxon test. [file Image_1.tif]

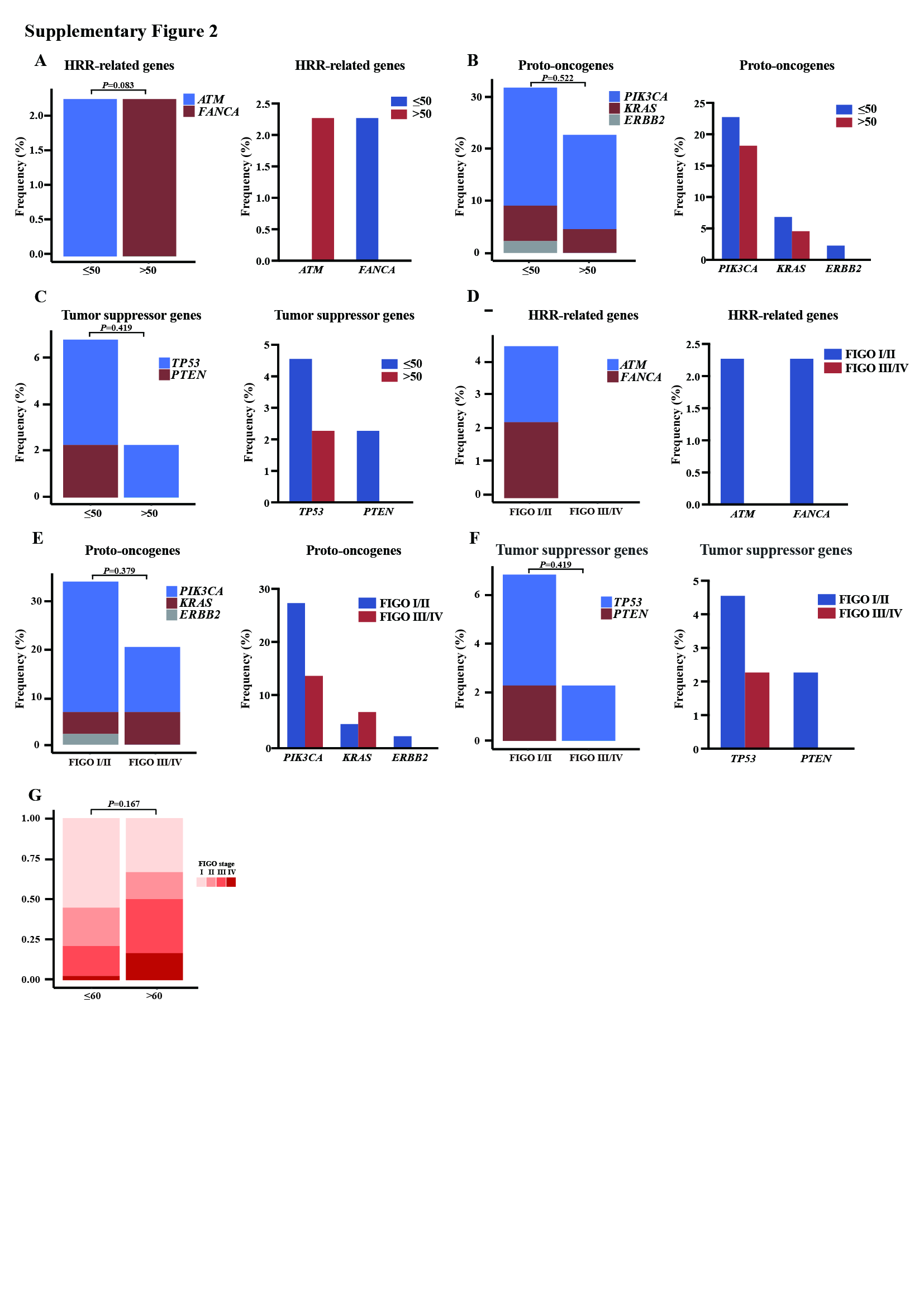

Supplement: Supplementary Figure 2 — Distribution of P/LP alterations according to age and FIGO stage in OCCC. The frequency of P/LP mutations in HRR-related genes (A), proto-oncogenes (B) or tumor suppressor genes (C) according to different age categories (≤ 50 years and > 50 years). The frequency of P/LP mutations in HRR-related genes (D), proto-oncogenes (E) or tumor suppressor genes (F) according to FIGO stage (FIGO I/II and FIGO III/IV). (A–F) were calculated by the Wilcoxon test. (G) The correlation between FIGO stage and different age categories (≤ 60 years and > 60 years). P values were calculated by the Kruskal-Wallis test. [file Image_2.tif]
